# Contrast Media-Induced Immune Hemolytic Anemia

**DOI:** 10.7759/cureus.14522

**Published:** 2021-04-16

**Authors:** Hajar H Al Ghailani, Abdullah M Al Alawi, Abdul Hakeem Al Hashim

**Affiliations:** 1 Medicine, Oman Medical Speciality Board, Muscat, OMN; 2 Medicine, Sultan Qaboos University Hospital, Muscat, OMN

**Keywords:** a radiographic contrast medium, immune-mediated hemolysis, hemolysis, hemolytic anemia, pneumonia

## Abstract

Drug-induced immune hemolytic anemia (DIIHA) is a rare type of immune-mediated hemolytic anemia, and mainly it is caused by antibiotics. There have been few case reports of contrast medium-induced immune hemolytic anemia. Here, we report a case of a 70-year-old woman who was admitted with community-acquired pneumonia. She had a CT abdomen and pelvis using iohexol (omnipaque), which resulted in severe hemolytic anemia and contributed to the patient’s death. This case illustrates a very rare complication of IV contrast medium that may result in death.

## Introduction

Drug-induced autoimmune hemolytic anemia (DIIHA) is relatively rare, may go undiagnosed in many cases, and the magnitude of hemolysis can vary widely [1]. Drugs may induce the formation of antibodies against red blood cell (RBC) membrane or intrinsic RBC antigen, which may induce immune-mediated hemolysis. The incidence of DIIHA is approximately 1 per million/year [2]. More than 130 drugs have been associated with DIIHA but the most commonly reported include cephalosporins, diclofenac, rifampicin, oxaliplatin, and fludarabine [3].

## Case presentation

A 70-year-old woman was brought to the emergency department at Sultan Qaboos University Hospital (SQUH) with a cough, shortness of breath, and fever of five days duration. Her medical background included ischemic stroke with severe disability (modified Rankin scale 5/6), type 2 diabetes mellitus, hypertension, depression, severe osteoarthritis, and atrial fibrillation. Her regular medications were quetiapine (25 mg BID), amlodipine (5 mg daily), esomeprazole (20 mg daily), glargine insulin (20 units at night), metformin (500 mg BID), bisoprolol (5mg daily), spironolactone (25 mg daily), and ferrous sulfate (200 mg daily).

On examination, the patient appeared sick and confused. Her vitals were as follows: temperature 37.2 °C, blood pressure 96/25 mmHg, pulse 130 beats per minute, respiratory rate 40/minute, and oxygen saturation 50% on ambient air. The chest examination revealed bilateral basal crackles, and on abdominal examination, she had a distended abdomen but no organomegaly. Other systemic examinations were unremarkable.

Laboratory findings are presented in Table 1. As summarized, the patient blood showed raised inflammation markers, deranged coagulation profile, raised lactate, acute kidney injury, and deranged liver functions.

**Table 1:** Important work-up results on the day of admission. WBC: white cell count; PT: prothrombin time; aPTT: activated partial thromboplastin time; INR: international normalized ratio.

Test	Admission day	Normal range
Hemoglobin (g/dL)	11.7	11-14.4
WBC (10^9^/L)	17.5	2.4-9.5
Neutrophil count (10^9^/L)	13.9	1-4.8
Platelets count (10^9^/L)	133	150-450
PT (second)	20.1	10.5-12.7
aPTT (second)	41	25.6-37.7
INR	1.83	0.92-1.08
Fibrinogen (g/L)	3.8	1.7-3.6
C-reactive protein	25	<5
Venous PH	7.2	7.35-7.45
Lactate (mmol/L)	7.7	0.5-1.6
Creatinine (mmol/L)	112	59-104
HCO_3_ (mmol/L)	22.5	22-24
Potassium (mmol/L)	7.2	3.5-5
Sodium (mmol/L)	144	135-145
Urea (mmol/L)	11.5	2.8-8.1
Alanine transaminase (U/L)	6658	0-33
Aspartate aminotransferase (U/L)	7771	0-17
Alkaline phosphatase (mmol/L)	107	35-104
Total bilirubin (µmol/L)	9	0-17
SARS-COV-2 PCR	negative	

**Figure 1:**
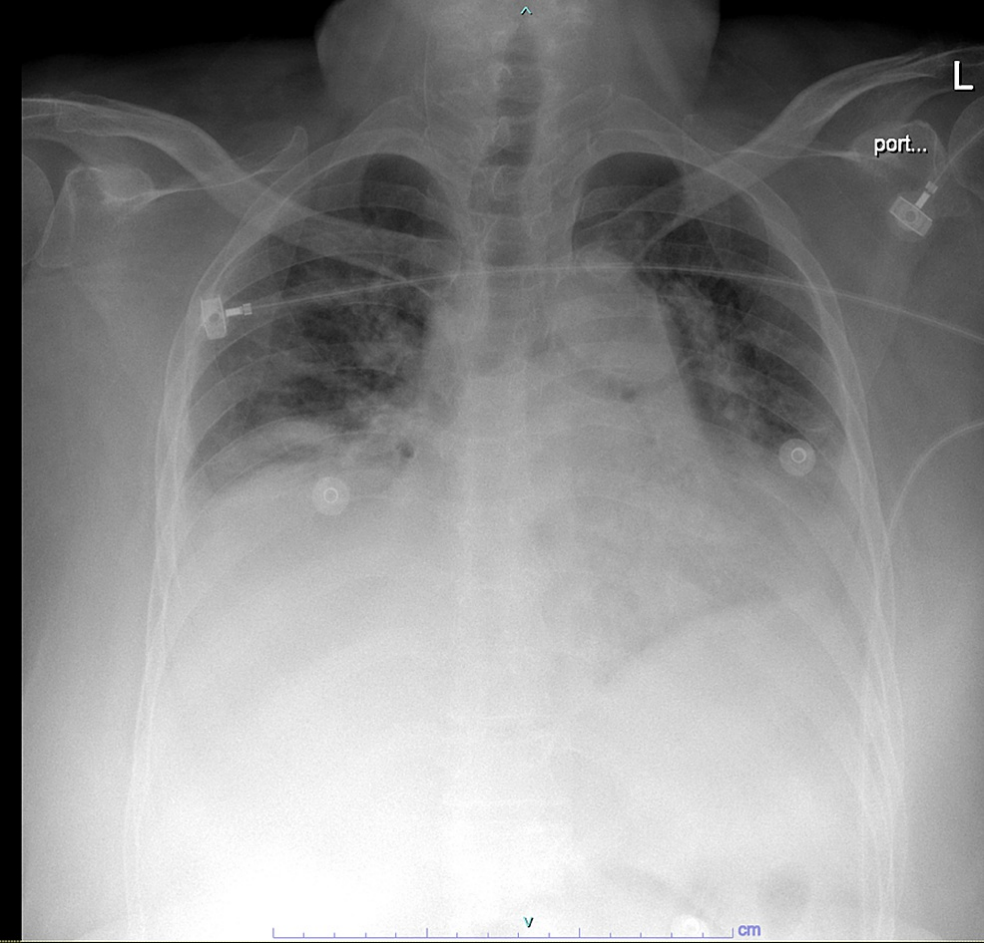
Chest X-ray shows bilateral pulmonary infiltrates and consolidation.

Chest X-ray demonstrated bibasilar infiltrates (Figure 1).

The patient was admitted with the impression of severe community-acquired pneumonia, causing septic shock, which led to multi-organ failure. The patient was intubated, mechanically ventilated, and started on IV antibiotics (piperacillin/tazobactam and azithromycin). She also required vasopressors for the management of her septic shock. Despite antibiotics, she remained febrile for the following 48 hours after; therefore, piperacillin/tazobactam was upgraded to meropenem. A CT scan of the abdomen and pelvis with IV contrast was done to look for an intraabdominal source of sepsis or hepatic vein thrombosis given the grossly deranged liver function test. The scan showed bilateral basal pulmonary consolidation and fatty liver without any other significant finding. Fourteen hours following the CT scan with IV contrast, her hemoglobin dropped from 10.6 to 7.6 g/dL (normal 11-14.4). There was no evidence of active bleeding. A blood film was performed, and it revealed normocytic normochromic red cells with anisocytosis, polychromia, bite cell, blister cells, acanthocytes, highly toxic neutrophils, and there were no fragmented cells. Other hemolytic workups were suggestive of active intravascular hemolysis (Table 2).

**Table 2:** Hemolytic work-up results. G6PD: glucose-6-phosphate dehydrogenase, SLA/LP: soluble liver antigen/liver-pancreas, LC-1: liver cytosol antigen type 1, AMA-M2: anti-mitochondrial M2 antibody, LKM-1: liver-kidney microsome type 1, gp210: glycoprotein-210, PML: promyelocytic leukemia.

	Result	Normal range
Reticulocyte count %	4.6	0.5-1.5
Lactate dehydrogenase (U/L)	5970	135-214
Haptoglobin (g/L)	<0.1	0.3-2
Platelets count (10^9^/L)	118	150-450
G6PD	40-70% activity	
aPTT (second)	46.1	25.6-37.7
PT (second)	16.4	10.5-12.7
INR	1.47	0.92-1.08
Fibrinogen (g/L)	3.9	1.7-3.6
Bilirubin	89	0-17
Direct anti-globulin test	Positive for IgG	
Mycoplasma serology	Negative	
Anti-SLA/LP	Negative	
Anti-LC-1	Negative	
AMA-M2	Negative	
Anti-Ro52	Negative	
Anti-LKM-1	Negative	
Anti-gp210	Negative	
Anti-PML	Negative	
Anti-Sp100	Negative	
3E (BPO)	Negative	

The patient was supported with blood transfusion, and despite that, unfortunately, she had a severe hemolysis course, which led to a further drop in her hemoglobin to 4 g/dL (normal 11-14.4). Her course was complicated by atrial fibrillation with a fast ventricular rate and acute kidney injury requiring renal replacement therapy. The patient’s condition deteriorated further, and she had cardiopulmonary arrest 48 hours after receiving IV contrast, and cardiopulmonary resuscitation was unsuccessful.

## Discussion

The patient was admitted with severe community-acquired pneumonia. Her intensive unit care admission was complicated by a severe course of hemolysis leading to patient deterioration. The differential diagnosis of hemolytic anemia in this clinical context included disseminated intravascular coagulopathy (DIC), glucose-6-phosphate dehydrogenase (G6PD) deficiency-induced hemolysis, and autoimmune hepatitis. However, normal fibrinogen level, blood film findings, negative autoimmune hepatitis screening largely excluded these differential diagnoses. The clinical presentation with cough and fever, deranged liver enzymes, and the positive direct anti-globulin test could be explained by the Ebstein Bar virus, which unfortunately was not tested. However, the early onset of severe hemolysis, absence of lymphocytosis or lymphopenia, and the absence of other clinical features, including inflamed throat, lymphadenopathy, hepatosplenomegaly, were against infective mononucleosis. Mycoplasma pneumonia can cause autoimmune hemolytic anemia; however, the clinical presentation and the negative mycoplasma serology test made this possibility unlikely [4]. The patient received piperacillin/tazobactam for the first 48 hours; however, hemolysis's abrupt onset followed the cessation of piperacillin/tazobactam made it an unlikely differential diagnosis.

The patient did not receive any of the medications which are known to be associated with DIIHA. It was evident that the patient developed a severe hemolysis course within hours after receiving 100 ml of iohexol (omnipaque) contrast, which makes contrast-induced immune intravascular hemolysis is the likely diagnosis [5].

Nonimmune hyperosmolar contrast-induced sickling and hemolysis were described in a patient with sickle cell disease following coronary angiography [6]. Only three cases of contrast medium induced immune intravascular hemolysis were reported in medical literature [2,7,8]. One patient developed severe hemolysis after the injection of Isopaque, an older ionic contrast medium, and serologic studies provided evidence for immunoglobulin (Ig)M, whereas the other two patients had intravascular hemolysis after administration of iomeprol contrast medium [2,8]. Drug-induced immune hemolysis (drug-dependent type) is the most likely explanation of the hemolysis in our case, evidenced by the abrupt severe hemolysis after the administration of iohexol (omnipaque), the positive DAT (IgG class), and the absence of other factors explaining the sever onset hemolysis. Cessation of the culprit drug and blood products transfusion are the main aspects in the management of drug-induced hemolytic anemia [9]. Steroids, intravenous immunoglobulins (IVIG), plasmapheresis, or immunosuppressants may be tried in refractory cases [10,11].

We report the first case report of severe immune-mediated hemolytic anemia precipitated by iohexol administration (omnipaque). Unfortunately, severe hemolytic anemia may have contributed to patient deterioration and death.

## Conclusions

This case reports the first case of iohexol (contrast media) induced immune hemolytic anemia in a critically sick patient. It highlights a very rare but serious complication of contrast medium. Contrast-induced immune hemolytic anemia should be known to clinicians and considered in the differential diagnosis of unexplained hemolysis after administration of contrast media.
